# CircRNA_15430 reduced by *Helicobacter pylori* infection and suppressed gastric cancer progression via miR-382-5p/ZCCHC14 axis

**DOI:** 10.1186/s13062-023-00402-9

**Published:** 2023-08-25

**Authors:** Yun Liu, Jia Cao, Qi Yang, Linqi Zhu, Wenjun Zhao, Xiuping Wang, Jun Yao, Yong Zhou, Shihe Shao

**Affiliations:** 1 Department of Digestive, the Affiliated People’s Hospital, Jiangsu University, No. 8 Dianli Road, Zhenjiang, Jiangsu 212002 China; 2 Endoscopy Center, Department of Gastroenterology, Shanghai East Hospital, School of Medicine, Tongji University, Shanghai, 200120 China; 3 Department of Pathology, the Affiliated People’s Hospital, Jiangsu University, Zhenjiang, Jiangsu 212002 China; 4 School of Medicine, Jiangsu University, 301 Xuefu Road, Zhenjiang, Jiangsu 212013 China

**Keywords:** circRNA_15430, *Helicobacter pylori*, Gastric cancer, Autophagy

## Abstract

**Background:**

*Helicobacter pylori* (*H.pylori, HP*) is one of the main causes of gastric cancer (GC). CircRNAs have been reported to play a crucial role in developing many types of cancer. However, the role of circRNAs in the development and progression of *HP* infected-GC has not been studied.

**Methods:**

The location of circRNA_15430 in GC cells were detected by nuclear and cytoplasmic RNA fractionation and RNA fluorescence in situ hybridization analysis (FISH) assays, and circRNA_15430, miR-382-5p and ZCCHC14 expression in GC cell lines and tissues were analyzed by quantitative real-time polymerase chain reaction (qRT-PCR). The function of circRNA_15430 in GC cells were examined by using colony formation, cell counting kit-8 (CCK-8) and Transwell assays, flow cytometry and laser scanning confocal microscopy. The protein levels were detected by Western blotting. Whether circRNA_15430 sponges miR-382-5p was monitored with a dual-luciferase reporter assay. Furthermore, circRNA_15430 was analyzed in vivo in tumor growth with nude mice.

**Results:**

CircRNA_15430 is primarily localized in the cytoplasm of GC cells, and downregulated in the GC cell lines and tissues, and is negatively correlated with the tumor size. Downregulation of circRNA_15430 promotes proliferation, migration and suppresses cell apoptosis and autophagy in GC cells. Mechanically, circRNA_15430 acts as a miR-382-5p sponge, alleviating the inhibitory effect of miR-382-5p on its target ZCCHC14. Knockdown circRNA_15430 enhances tumor growth *in vivo.* In addition, circRNA_15430 was reduced in *HP* + gastritis tissues and *HP*-infected MGC-803 cells, reversing the pro-*HP* effect on autophagy. Additionally, miR-382-5p was increased in *HP* + gastritis tissue and *HP*-infected MGC-803 cells while ZCCHC14 decreased in *HP*-infected MGC-803 cells. MiR-382-5p reverses the effect of si-ZCCHC14 on autophagosome numbers in MGC-803 cells.

**Conclusions:**

Therefore, circRNA_15430 plays an inhibitory role in GC and regulates the progression of *HP* infection-related GC, providing a novel molecular marker for GC therapy.

**Supplementary Information:**

The online version contains supplementary material available at 10.1186/s13062-023-00402-9.

## Introduction

Gastric cancer (GC) is an important cancer worldwide that was responsible for more than one million new cases and an estimated 769,000 deaths in 2020, ranking fifth in incidence and fourth in mortality globally [1]. GC is a multifactorial disease, and an infection of *HP* and environmental and genetic factors can lead to GC progression. The high aggressiveness and early stomach problems lack obvious symptoms of GC, resulting in rapid progression [2–4].

Several studies found that noncoding RNAs could mediate *HP* to regulate the proliferation and cell apoptosis of gastric epithelial cells. Zhang et al. found that LncRNA H19 expression was upregulated in *HP*-infected GC tissues and cells. Moreover, H19 promoted the proliferation, migration and invasion of *HP*-infected GC cells by enhancing NF-κB-induced inflammation [5]. Ali Rajabi et al. reported that lncRNA HOXA-AS2 was elevated significantly in cancerous tissues and related to *HP* infection [6]. Furthermore, lncRMA HOTAIR, FOXD2-AS1, AF147447 and OC1 play important role in *HP* infection associated GC [7–10]. In addition, miRNAs plays a significant role in *HP-*infected GC cells [11–13].

On the other hand, with the development of high-throughput sequencing technology, circRNA are attracting the increasing attention of researchers. CircRNA have a highly conserved and stable covalently closed cyclic structure that is abundant and stable in eukaryotic cells and can resist RNase R enzyme digestion [14–16]. CircRNAs can regulate the disease progression through sponging miRNAs, encoding amino acids, and regulating mRNA stability and so on [17–19]. Guo et al. showed that *HP* could upregulate circMAN1A2 expression in AGS and BGC-823 cells independently of CagA. The downregulation of circMAN1A2 could inhibit the proliferation, migration, and invasion of GC cells and could promote the progression of GC induced by *H.pylori* by sponging miR-1236-3p to regulate MTA2 expression [20]. Zhang et al. reported that in early gastric cancer after endoscopic submucosal dissection, circFNDC3B could target miR-942 and miR-510 and mediate the expression of CD44 and CDH1. In addition, a *HP* infection could promote the expression of circFNDC3B, which also resulted in upregulated CD44 and CDH1 mRNA levels in rTip-α cultivated MKN28 cells [21]. Many studies have examined the expression and role of circRNAs in GC, but there have been few studies on circRNAs in *HP* infection-related GC.

In this study, we analyzed the Gene Expression Omnibus (GEO) database (http://www.ncbi.nlm.nih.gov/gds/) (GSE100170, GSE152309, GSE163416 and GSE131414) demonstrated that has_circ_00015430 (named circRNA_15430) that localized to *ABL2* gene may play a significant role in GC progression, and found it was down-regulated in GC tissues and cell lines. Knocked-down circRNA_15430 expression could promote the ability of cell proliferation and migration and inhibit cell apoptosis and autophagy in GC cells. Furthermore, circRNA_15430 sponged the miR-382-5p to regulate the expression of ZCCHC14 and influence the progression of GC. In addition, we also found that the expression of circRNA_15430 was decreased in *HP* + gastritis tissues and MGC-803 cells infected with *HP*, and the low expression of circRNA_15430 could reverse the autophagy promoted by *HP* infections.

## Materials and methods

### Patients and specimens

GC tissues and their corresponding paracancer tissues (≥ 5 cm from tumor edge), from 42 patients who underwent surgical treatment for GC from October 2019 to December 2021 were obtained for RNA extraction. Inclusion criteria: patients who had not received any form of anti-tumor therapy, such as chemoradiotherapy before surgery, and were diagnosed with GC after a pathological diagnosis. Exclusion criteria: patients with a family history of GC and those who had received radiotherapy or chemotherapy. The GC patients included 14 females and 28 males, the median age of patients is 69.93 ± 8.303 years. Detailed clinicopathological data of these GC patients are shown in Table 1.

Nineteen *HP* − human gastritis specimens and twelve *HP* + human gastritis specimens were collected from patients undergoing gastroscopy, and gastritis tissues were used to detect *HP* infections by a rapid urease test.

All patients’ tissues were obtained from The Affiliated People’s Hospital of Jiangsu University. This study was approved by the Medical Ethics Committee of Jiangsu University, and all participants signed an informed consent form.

**Table 1:** The correlation of circRNA_15430 expression with the clinical features of gastric cancer (GC) patients

	Low (n = 21)	High (n = 21)	*P* value
Gender			
Male	13	15	0.7442
Female	8	6	
Age			
≤ 60	3	5	0.6965
> 60	18	16	
Tumor size			
≤ 5	6	14	0.0284*
> 5	15	7	
LNM			
yes	13	10	0.5359
no	8	11	
TNM			
I+II	4	8	0.3058
III+IV	17	13	

*P < 0.05

### Cell lines, *HP* strains and the infection model

GES-1 human gastric epithelial cells and GC cell lines MGC-803, BGC-823, and HGC-27 were cultured according to the methods described in a previous article [22]. The *HP* strain 26,695 (obtained from Marshall Digestive Disease International Center, Shanghai East Hospital) was grown on Columbia Agar Base (Autubio Co., LTD, Zhengzhou, Henan, China) at 37 °C under microaerophilic conditions (5% O_2_, 10% CO_2_, and 85% N_2_). *HP* was suspended in RPMI-1640 medium without antibiotics and the number of bacteria was determined by measuring optical density at 600 nm (1 OD 600 = 1 × 10^8^ CFU/mL). RPMI-1640 medium alone served as a blank control. The cultured cells were seeded on plates and grown to 80% confluence. Subsequently, the MGC-803 cells were infected with *HP26695* for 4, 8 and 12 h at a multiplicity of infection (MOI) of 50:1. *HP26695* was then used to infect MGC-803 cells for 4 and 8 h at MOIs of 50:1 and 100:1.

### RNase R treatment

RNase R enzyme (BioVision Technologies, LCC, San Francisco, USA) was used to purify the MGC-803 cell RNA at 37℃ for 30 min to remove the linear RNA and incubated at 70℃ for 10 min to inactivate the enzyme. The expression was detected by PCR and analyzed by agarose electrophoresis.

### Fluorescence in situ hybridization analysis (FISH) assay

The Cy3 labeled probe for circRNA_15430 (the sequence is TCAGCAGTCACA + TACACCTAAAAGT + TGTA) was designed and synthesized by Suzhou GenePharma Co., Ltd (Suzhou, China). The RNA-FISH kit was procured from Gene Pharma and the methods described in a previous article [22].

### RNA extraction and qRT-PCR

The RNA isolater, Total RNA Extraction Reagent (Nanjing Vazyme Biotechnology Co., LTD, Nanjing, China) was used to extract the total RNA from cells and tissues. Nuclear and cytoplasmic RNAs were extracted using NE-PER Nuclear and Cytoplasmic Extraction Reagents (Thermo Scientific, USA) according to the.

Manufacturer’s protocol. HiScript III 1st Strand cDNA Synthesis Kit (+ gDNA wiper) (VazymeD) reversed transcription of RNA into cDNA. QRT-PCR was conducted using AceQ qPCR SYBR Green Master Mix (High ROX Premixed) (Vazyme) and Q3. All primers used are listed in Additional file 1: Table S1.

### Small interfering RNA (siRNA), plasmid and lentivirus transfection

The circRNA_15430 siRNAs, miR-382-5p mimics, inhibitor, and ZCCHC14 siRNAs were all designed and purchased from Suzhou GenePharma Co., Ltd (Suzhou, China). According to the sequence across the back-splicing junction site of circRNA_15430, circRNA_15430 siRNAs were designed to knock down the circRNA_15430 expression. The pcicR-circRNA_15430 plasmid, PmirGLO-circRNA_15430-WT, and PmirGLO-circRNA_15430-MUT were purchased from Sangon Biotech Co., Ltd (Shanghai, China). Autophagy plasmid pcDNA-eGFP-LC3 was stored in the laboratory. The siRNA and plasmids were transfected with Lipofectamine 3000 (ThermoFisher Scientific, USA).

The sequence of siRNAs were listed as follows:

si-circRNA_15430: 5’-CAACUUUUAGGUGUAUGUGTT-3’.

5’-CACAUACACCUAAAAGUUGTT-3’.

si-ZCCHC14: 5’-GGAAACUGCGUUUGCACAATT-3’.

5’-UUGUGUAAACGCAGUUUCCTT-3’.

### Colony formation and CCK-8 assay

Approximately 1 × 10^3^ transfected cells were seeded into a six-well plate and incubated. The medium was changed at three-day intervals. After 10–14 days of incubation, the cells were fixed with 4% paraformaldehyde and stained with crystal violet. Approximately 1 × 10^3^ cells were seeded in 96-well plates and cultured. Next day, 10 µL of the Cell Counting Kit-8 (CCK-8) reagent (Tongren, Shanghai, China) was added to the wells to be detected. After 1 h of culture, the absorbance was detected at 450 nm using a microplate reader for different times.

### Transwell assays

CoStar Transwell chambers (8 μm pore size; Corning, Costar, NY, USA) and 2 × 10^4^–1 × 10^5^ cells in 300 µL of serum-free media were added to the upper chambers, while the lower chambers were filled with 600 µL of medium containing 10% FBS to induce cell migration. After 12-24 h of incubation, the cells that migrated to the lower surface of the membrane were fixed with 4% paraformaldehyde and stained with crystal violet.

### Flow cytometry

The apoptosis kit was purchased from Vazyme. The cells were collected, washed with ice-cold PBS, and resuspended in 500 µL of 1× binding buffer. Subsequently, 5 µL of an annexin V-fluorescein isothiocyanate (FITC) staining solution containing 5 µL of PI was added to the suspension. The treated cells were analyzed using a BD FACS Calibur flow cytometer (Becton–Dickinson, Franklin Lakes, NJ, USA). Three independent experiments were performed.

### Western blotting

The cells were washed with cold PBS, and the protein lysates RIPA, protease inhibitors and phosphatase inhibitors were added to the cells at a ratio of 100:1:1. The cells were lysed on ice for 45 min-1 h, and centrifuged at 13,000 rpm/min for 30 min to obtain the total cell protein. Subsequently, 200 µg of cellular protein was isolated on SDS-Page gel (10%) and transferred to a PVDF membrane. The PVDF membrane was sealed with 5% skim milk for 1-2 h at room temperature, and the antibodies (Additional file 2: Table S2) were incubated overnight on a shaker at 4℃. The next day, HRP labeled secondary antibody was incubated for 1 h at room temperature. After the membrane was washed, an ECL luminescent reagent was used to take pictures, GAPDH was used as an internal reference, and image J software was used to analyze the gray values of each strip. Target protein expression level = target protein grayscale value /GAPDH grayscale value.

### Laser scanning confocal microscope

MGC-803 cells were divided into si-circRNA_15430 and NC group and co-transfected with autophagy plasmid pcDNA-eGFP-LC3. Subsequently, the cells were treated with 1 µg/mL rapamycin for 18 h; 1 × 10^4^ cells were then inoculated on cell glass coverslips. The next day, after being fixed with 4% paraformaldehyde for 30 min at room temperature, the slivers were washed five times with PBS at room temperature for 5 min each. Hoechst 33,258 was stained at room temperature for 8 min, and washed five times with PBS for 5 min each. The slivers were removed and sealed with anti-fluorescence-quenching sealing tablets. The number of autophagosomes in each group was photographed using a laser confocal microscope.

### Dual-luciferase reporter assay

The luciferase reporter plasmid pmirGLO-luc2 containing the wild-type circRNA_15430 (WT) or mutant circRNA_15430 (MUT) in the binding sites of miR-382-5p was constructed and synthesized by GenePharma Co. (Suzhou, China). The luciferase reporter plasmids were then co-transfected into 293T cells with NC or miR-382-5p mimics using Lipofectamine™ 3000 reagent (ThermoFisher Scientific). After 24–36 h incubation, the firefly luciferase and renilla luciferase activity were detected. The luciferase reporter plasmids were calculated from the firefly luciferase/ Renilla luciferase activity ratio to determine if there were binding sites between circRNA_15430 and miR-382-5p. All experiments were repeated three times.

### Animal experiments

Four-week-old male nude mice were purchased and housed in the Model Animal Research Center of Jiangsu University. All experimental procedures were performed according to the regulations and internal biosafety and bioethics guidelines of Jiangsu University and the Jiangsu Municipal Science and Technology Commission (UJS-IACUC-AP-2021030404). A total of 2 × 10^6^ MGC-803 cells (NC and sh-circRNA_15430 group; n = 5/group) and 2 × 10^6^ BGC-823 cells (Vector and OE-circRNA_15430 group; n = 5/group) per mouse were resuspended in sterile PBS and injected into the backs of nude mice. The length and width of the tumor were measured using Vernier calipers once every three days and recorded for three weeks, calculated tumor volumes (V = 1/2×L×W^2^). Three-four weeks later, the tumor was removed and weighed.

### Statistical analysis

Statistical analysis was conducted using Graphpad 8.0. All data were presented as the mean ± SD of at least three biological replicates. The student’s t-test and Mann–Whitney U test are generally used to analyze the differences between the two groups. ANOVA was used to analyze the samples among multiple groups. *P* < 0.05 was considered to indicate a significant difference.

## Results

### CircRNA_15430 is down-regulated in gastric cancer tissues and cell lines

**Fig. 1:**
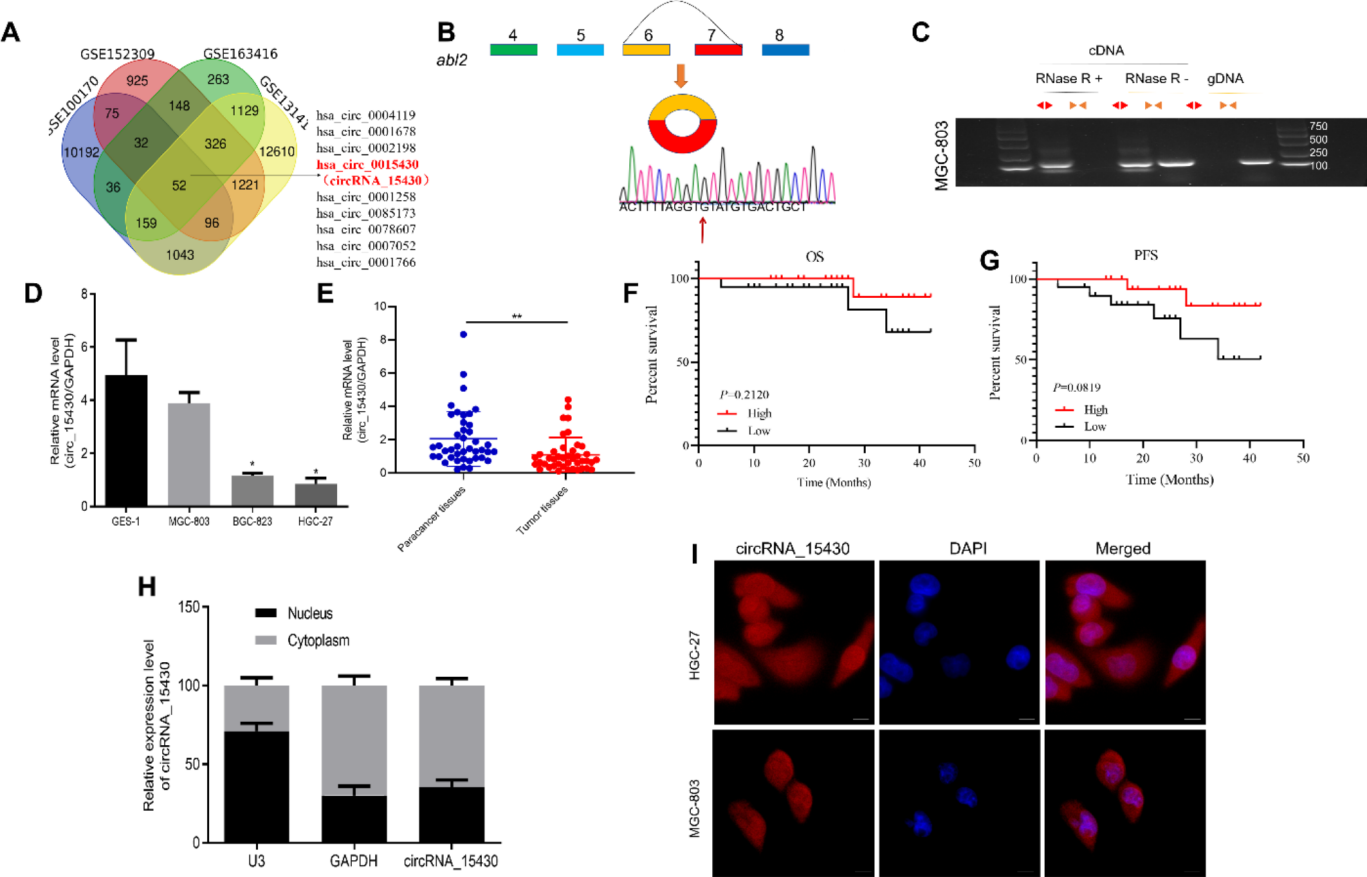
Expression of circRNA_15430 is low in GC cell lines and tissues. **A** GEO dataset (GSE100170, GSE152309, GSE163416 and GSE131414) were downloaded for analyzing abnormal expression of circRNAs in GC tissues. **B** Schematic illustration of the formation of circRNA_15430 via the circularization of exons in the *abl2* gene. PCR product sequencing conformed to the head-to-tail splicing of circRNA_15430. **C** Gel electrophoresis validated the existence of the back-spliced and canonical forms of *abl2* in the presence or absence of RNase R enzyme supplementation in cDNA and gDNA from GC cells. **D** qRT-PCR detected circRNA_15430 expression in GES-1 cells and three GC cell lines. **E** qRT-PCR detected relative circRNA_15430 level in 42 paired fresh normal gastric tissues and GC tissues. **F** and **G** The correlation between OS and PFS with circRNA_15430 expression in GC patients. **H** and **I** Nuclear-cytoplasmic fractionation and RNA FISH assay found that circRNA_15430 is located both in the cytoplasm and nucleus of GC cells (scale bars = 25 μm). *P < 0.05, **P < 0.01

To identify new circRNAs in GC, we extracted circRNA microarray data (GSE100170, GSE152309, GSE163416 and GSE131414) from GEO databases to analyze circRNA expression profiles, and found circRNA_15430 maybe abnormally expressed in GC tissues (Fig. 1A). Considering the relative expression level and detection specificity, we chose hsa_circ_0015430 as the target for further study. Hsa_circ_0015430, also named circRNA_15430, was spliced from exons 6 and 7 of the *abl2* gene and formed an antisense-overlapping circular transcript of 358 nucleotides. The PCR products detected the cyclization sites, and it was a head-to-tail spliced product (Fig. 1B). Specifically designed divergent and convergent primers with PCR and an agarose gel electrophoresis assay detected the expression levels of back-spliced and canonical forms of ABL2 with or without RNase R supplementation in MGC-803 cells cDNA and gDNA. The results showed that circRNA_15430 could resist digestion by RNase R (Fig. 1C). QRT-PCR showed that, compared to GES-1 cells, circRNA_15430 has a lower expression in GC cells lines (Fig. 1D). In addition, compared to corresponding paracancer tissues, the expression level of circRNA_15430 was lower in the GC tissues of 42 GC patients (Fig. 1E). The expression of circRNA_15430 was correlated with tumor sizes in GC patients (Table 1). Also, there is no significant correlation between the overall survival (OS) and progression-free survival (PFS) with circRNA_15430 expression in GC patients (Fig. 1F and G). Furthermore, nuclear and cytoplasmic RNA fractionation and RNA FISH assay suggested that circRNA_15430 was localized both in the cytoplasm and nucleus of GC cells, and mainly in the cell cytoplasm (Fig. 1H and I).

### CircRNA_15430 could suppress the proliferation and migration and promote the apoptosis of gastric cancer cells

**Fig. 2:**
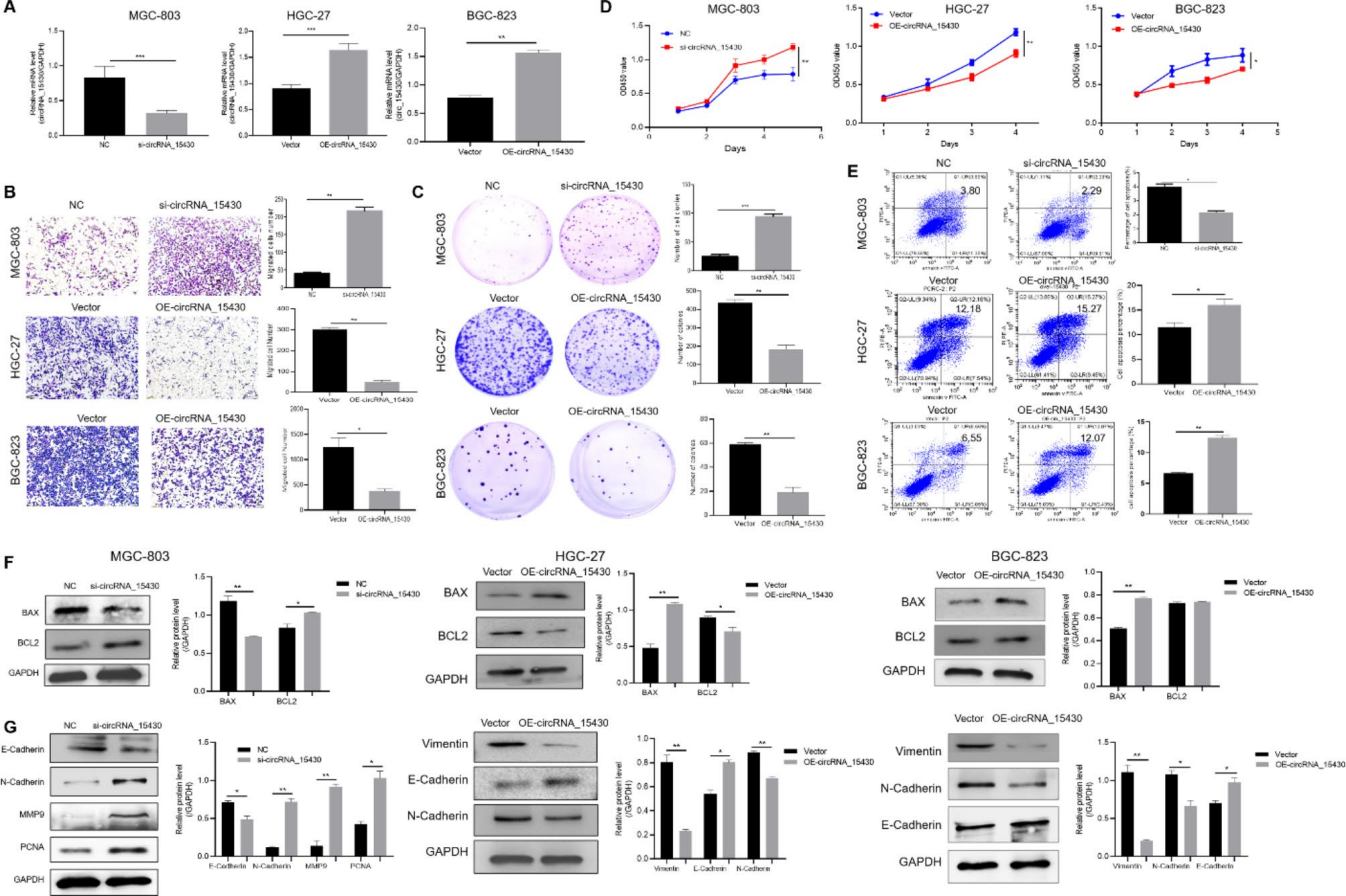
CircRNA_15430 over-expression inhibits migration and growth, and promotes cell apoptosis percentage in GC cells. **A** qRT-PCR detected the knockdown and over-expression efficiency in GC cells **B** Transwell migration assay (scale bars = 200 μm), **C** colony formation assay, **D** CCK-8 assay, and **E** flow cytometry assay was used to analyze the effects of abnormal circRNA_15430 expression on metastasis, proliferation, and cell apoptosis of GC cells. **F** and **G** Western blotting examined the expression levels of BAX, BCL2, and EMT-related proteins in MGC-803 cells transfected with NC and si-circRNA_15430, HGC-27 cells and BGC-823 cells transfected with Vector and circRNA_15430 over-expression plasmids. *P < 0.05, **P < 0.01, ***P < 0.001

MGC-803 cells were selected to knock down circRNA_15430 expression with siRNA that targeted the cyclization site. The HGC-27 cells and BGC-823 cells were transfected with the plasmids to overexpress circRNA_15430 for functional verification in vitro (Fig. 2A). Transwell migration assay suggested that high expression of circRNA_15430 could inhibit the migration of GC cells (Fig. 2B). In addition, colony formation and CCK-8 assay demonstrated that high circRNA_15430 suppressed the proliferation of GC cells (Fig. 2C and D). In addition, flow cytometry showed that the downregulation of circRNA_15430 may suppress the percentage of cell apoptosis in MGC-803 cells (Fig. 2E). Western blotting found that knock down circRNA_15430 in MGC-803 cells decreased the expression of BAX and E-Cadherin, while increasing the expression of BCL2, N-Cadherin, MMP2, and PCNA. BAX and E-Cadherin expression were increased when circRNA_15430 was overexpressed in HGC-27 cells and BGC-823 cells, while Vimentin and N-Cadherin expression were decreased. Furthermore, BCL2 was decreased in HGC-27 cells while BGC-823 cells showed no obvious change (Fig. 2F and G).

### CircRNA_15430 could act as a miR-382-5p sponge in GC cells

**Fig. 3:**
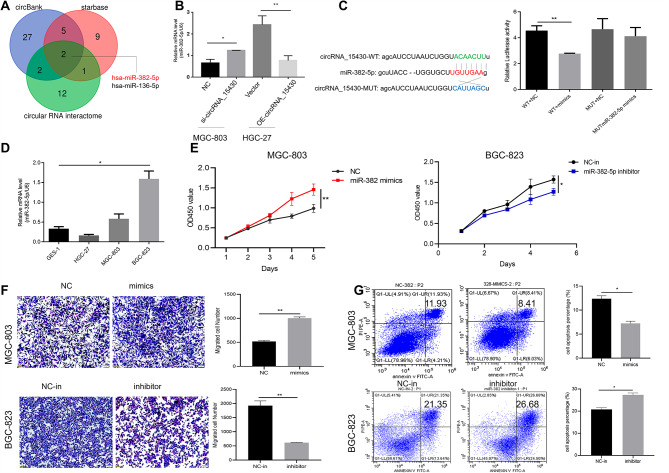
CircRNA_15430 could act as a miR-382-5p sponge in GC cells. **A** Three public database predicts the miRNAs could be sponged by circRNA_15430. **B** qRT-PCR detected the level of miR-382-5p expression when abnormal expression of circRNA_15430 in GC cells. **C** The dual-luciferase assay detected the luciferase activity when luc-circRNA_15430-WT, and luc-circRNA_15430-MUT co-transfected with miR-382-5p mimics in 293T cells. **D** The expression level of miR-382-5p in GES-1 cells and GC cells was detected by qRT-PCR. **E** CCK-8 assay, **F** Transwell migration assay, and **G** Flow cytometry detected the function of miR-382-5p in GC cells. *P < 0.05, **P < 0.01

Experiments were conducted to examine how circRNA_15430 mediates the proliferation, migration, and cell apoptosis in GC cells. RNA FISH and nuclear and cytoplasmic RNA fractionation suggested that circRNA_15430 was localized both in the cytoplasm and nucleus of GC cells and mainly in the cell cytoplasm. CircRNA_15430 might be involved in the function of GC cells by absorbing miRNA through sponging action. Therefore, circBANK, starbase and circular RNA interactome public database were used to predict the miRNAs that might bind to circRNA_15430, found that miR-382-5p and miR-136-5p had a stronger binding ability to circRNA_15430 (Fig. 3A). QRT-PCR detected the expression levels of the two miRNAs and found that the expression of miR-382-5p was increased after knockdown of circRNA_15430 in MGC-803. By contrast, the overexpression of circRNA_15430 in HGC-27 cells resulted in a decrease in miR-382-5p (Fig. 3B). A dual-luciferase reporter with WT circRNA_15430 or MUT circRNA_15430 in the binding sites of miR-382-5p was constructed, and miR-382-5p mimics were co-transfected with the luciferase reporter plasmids into 293T cells. Compared to the control, a significant decrease in the luciferase ratio was observed when the cells were co-transfected with miR-382-5p mimics (Fig. 3C). In addition, miR-382-5p was highly expressed in BGC-823 cells and low in HGC-27 cells and MGC-803cells (Fig. 3D). Related experiments showed that the miR-382-5p mimics promoted cell migration and proliferation and inhibited the percentage of cell apoptosis in MGC-803 cells while the miR-382-5p inhibitor could suppress cell migration and proliferation while enhancing the percentage of cell apoptosis in BGC-823 cells (Fig. 3E-G). Hence, circRNA_15430 could sponge absorb miR-382-5p in GC cells.

### MiR-382-5p reverses the ability of circRNA_15430 to suppress GC progression

**Fig. 4:**
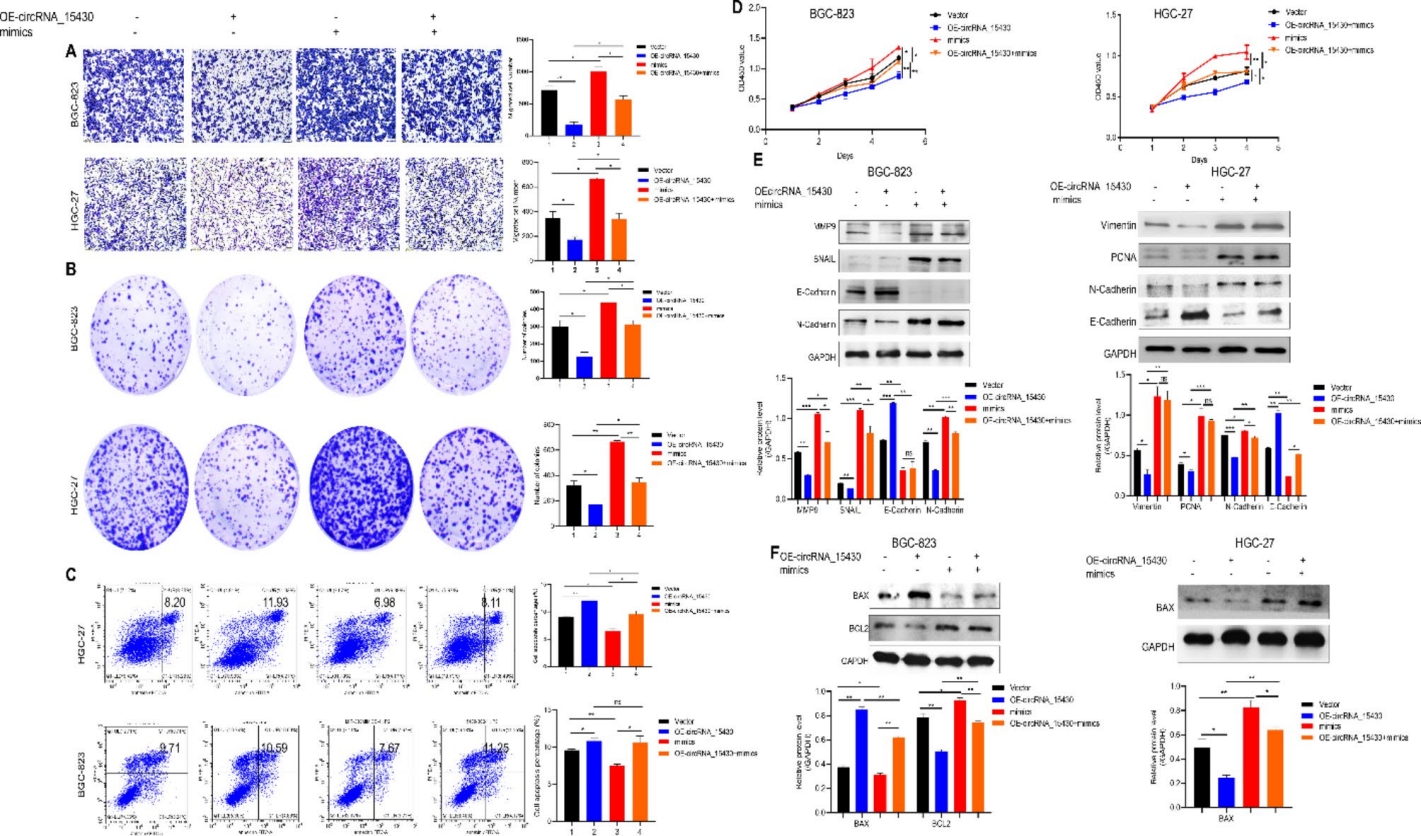
CircRNA_15430 inhibited migration and proliferation, and promoted cell apoptosis in GC cells by regulating miR-382-5p. **A** Transwell migration assay, **B** colony formation assay, **C** Flow cytometry assay, **D** CCK-8 assay detected the effect of circRNA_15430 over-expression plasmids co-transfected with miR-382-5p mimics in BGC-823 and HGC-27 cells on migration, proliferation, and cell apoptosis. **E** and **F** Western blotting detected EMT and cell apoptosis-related proteins expression levels when circRNA_15430 over-expression plasmids co-transfected with miR-382-5p mimics in BGC-823 and HGC-27 cells. *P < 0.05, **P < 0.01, ***P < 0.001

Rescue experiments of abnormal expression of miR-382-5p and circRNA_15430 were performed to determine whether circRNA_15430 could exert a biological function by sponging miR-382-5p. Migration, colony formation, CCK-8. and cell apoptosis assays were conducted. The results showed that the miR-382-5p mimics attenuated the ability of circRNA_15430, suppressed the proliferation and migration of GC cells and promoted the percentage of cell apoptosis in HGC-27 cells and BGC-823 cells (Fig. 4A-D). The miR-382-5p mimics could reverse the related protein levels such as MMP9, SNAIL, Vimentin, PCNA, E-Cadherin, N-Cadherin, BAX, and BCL2 that were altered by OE-circRNA_15430 in HGC-27 cells and BGC-823 cells (Fig. 4E and F). In summary, circRNA_15430 suppresses GC progression by sponging miR-382-5p in GC cells.

### ZCCHC14 is a direct target of miR-382-5p and acts as a tumor-inhibiting factor in GC

**Fig. 5:**
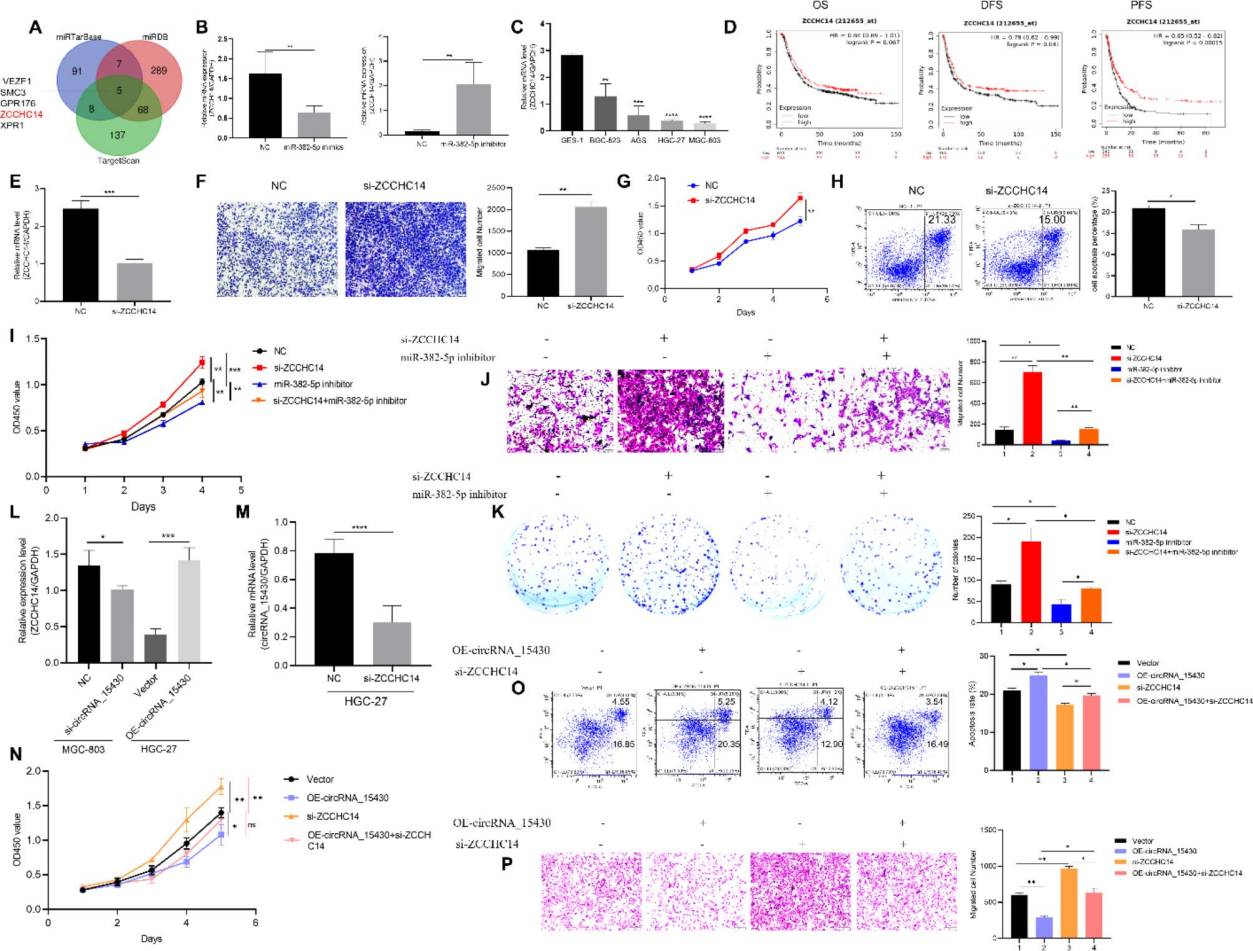
MiR-382-5p targets ZCCHC14 and regulates the progression of gastric cancer. **A** Three public database predicted the genes targeted by miR-382-5p. **B** qRT-PCR detected the effect of abnormal miR-382-5p expression on the expression of ZCCHC14 in GC cells. **C** The expression levels of ZCCHC14 in GES-1 cells and four GC cells were detected by qRT-PCR. **D** Kaplan-Meier Plotter database was used to analyze the relationship between ZCCHC14 expression and OS, DFS, and PFS in GC patients. **E** qRT-PCR was used to detect the effect of si-ZCCHC14 on the expression of ZCCHC14 in HGC-27 cells. **F** Transwell migration assay, **G** CCK-8 assay, **H** Flow cytometry assay detected the effect of ZCCHC14 knockdown on migration, growth and cell apoptosis in HGC-27 cells. **I** CCK-8 assay, **J** Transwell migration assay, and **K** colony formation assay detected the effect of si-ZCCHC14 and miR-382-5p inhibitor co-transfected into HGC-27 cells on cell migration and proliferation. **L** qRT-PCR detected the expression of ZCCHC14 when knockdown and overexpress circRNA_15430 in GC cells. **M** qRT-PCR detected the expression of circRNA_15430 when knockdown ZCCHC14 in HGC-27 cells. **N** CCK-8 assay, **O** flow cytometry assay, and **P** Transwell migration assay examined the function of co-transfection of circRNA_15430 plasmids and ZCCHC14 siRNA in HGC-27 cells on cell growth, apoptosis and metastasis. *P < 0.05, **P < 0.01, ***P < 0.001, ****P < 0.0001

TargetScan, miRTarBase and miRDB predicted the direct target of miR-382-5p and found that five genes that it might regulate (Fig. 5A). The influence of abnormal expression of miR-382-5p on the predicting target gene expression was detected by qRT-PCR, which suggested that miR-382-5p mimics reduced the ZCCHC14 expression level and miR-382-5p inhibitor enhanced its expression in GC cells (Fig. 5B). Furthermore, compared to GES-1 cells, the mRNA expression of ZCCHC14 was decreased in GC cells (Fig. 5C). The public database Kaplan-Meier Plotter (http://kmplot.com/) was used, and a high expression of ZCCHC14 was found in GC patients, which predicts poor OS, disease-free survival (DFS), and PFS (Fig. 5D). ZCCHC14 target siRNA was then used to knock down its expression in HGC-27 cells (Fig. 5E) and found that it could promote cell migration, proliferation and suppress the percentage of cell apoptosis (Fig. 5F-H). Furthermore, the results also showed that the miR-382-5p inhibitor could reverse the effects of ZCCHC14 si-RNA on the metastasis and proliferation of HGC-27 cells (Fig. 5I-K). The expression of ZCCHC14 was decreased in MGC-803 cells when knocked down circRNA_15430 and increased in HGC-27 cells that overexpressed circRNA_15430 (Fig. 5L). While, the knockdown of ZCCHC14 in HGC-27 cells decreased the expression of circRNA_15430 (Fig. 5M). In addition, the siRNA targeted ZCCHC14 could reverse the effects of circRNA_15430 plasmids on the growth, cell apoptosis and migration in HGC-27 cells (Fig. 5N-P). These results suggested that circRNA_15430 could mediate the development of GC through the miR-382-5p/ZCCHC14 axis.

### High expression of circRNA_15430 inhibits the proliferation of GC cells in vivo

**Fig. 6:**
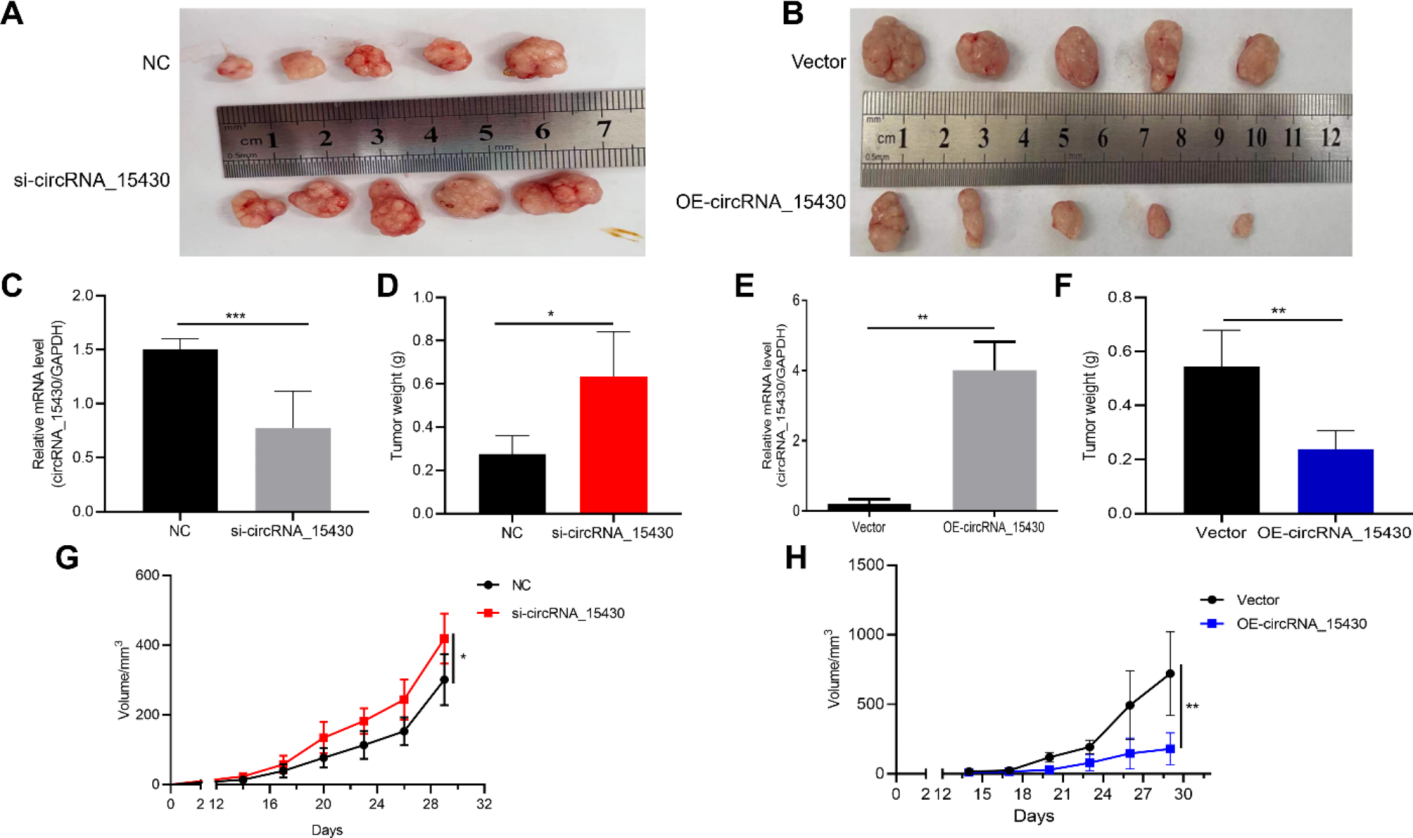
circRNA_15430 knockdown promoted xenograft tumour growth in vivo. **A** MGC-803 cells knockdown circRNA_15430 and NC group were used to obtain the tumours of the xenograft mouse model and image. **B** BGC-823 cells which overexpressed circRNA_15430 and Vector group to analyzed the effect on the subcutaneous tumor in nude mice. **C** qRT-PCR detected the level of circRNA_15430 expression in NC and si-circRNA_15430 group subcutaneous tumor tissues. **D** Tumor weight of NC and si-circRNA_15430 group subcutaneous tumor. **E** qRT-PCR detected the expression level of circRNA_15430 in Vector and OE-circRNA_15430 group in BGC-823 cells in subcutaneous tumor tissues. **F** Tumor weight of Vector and OE-circRNA_15430 group subcutaneous tumor. **G** and **H** The tumor volume of each group were analyzed. *P < 0.05, **P < 0.01, ***P < 0.001

Previous studies showed that high expression of circRNA_15430 could significantly inhibit the proliferation of gastric cancer cells in vitro. Therefore, a xenograft mouse model was established to detect the effects of circRNA_15430 on the growth of GC in vivo. Compared with the NC group, the MGC-803 cells that knockdown circRNA_15430 expression increased tumor growth significantly (Fig. 6A). In addition, BGC-823 cells with high circRNA_15430 expression suppress tumor growth (Fig. 6B). Furthermore, the expression of circRNA_15430 decreased in the knockdown group mouse tumor tissues (Fig. 6C). The weight (Fig. 6D) and volume (Fig. 6G) of the tumors tissues in this group were obviously lower than that in the NC group. Compared with the Vector group, however, circRNA_15430 expression was increased in the overexpression group (Fig. 6E). The weight (Fig. 6F) and volume (Fig. 6H) of the tumor tissues in the circRNA_15430 overexpression group were increased significantly. These results suggested that the downregulation of circRNA_15430 could promote the growth of GC cells in vivo.

### Down-regulation of circRNA_15430 reduced *HP* infection-induced autophagy in MGC-803 cells

**Fig. 7:**
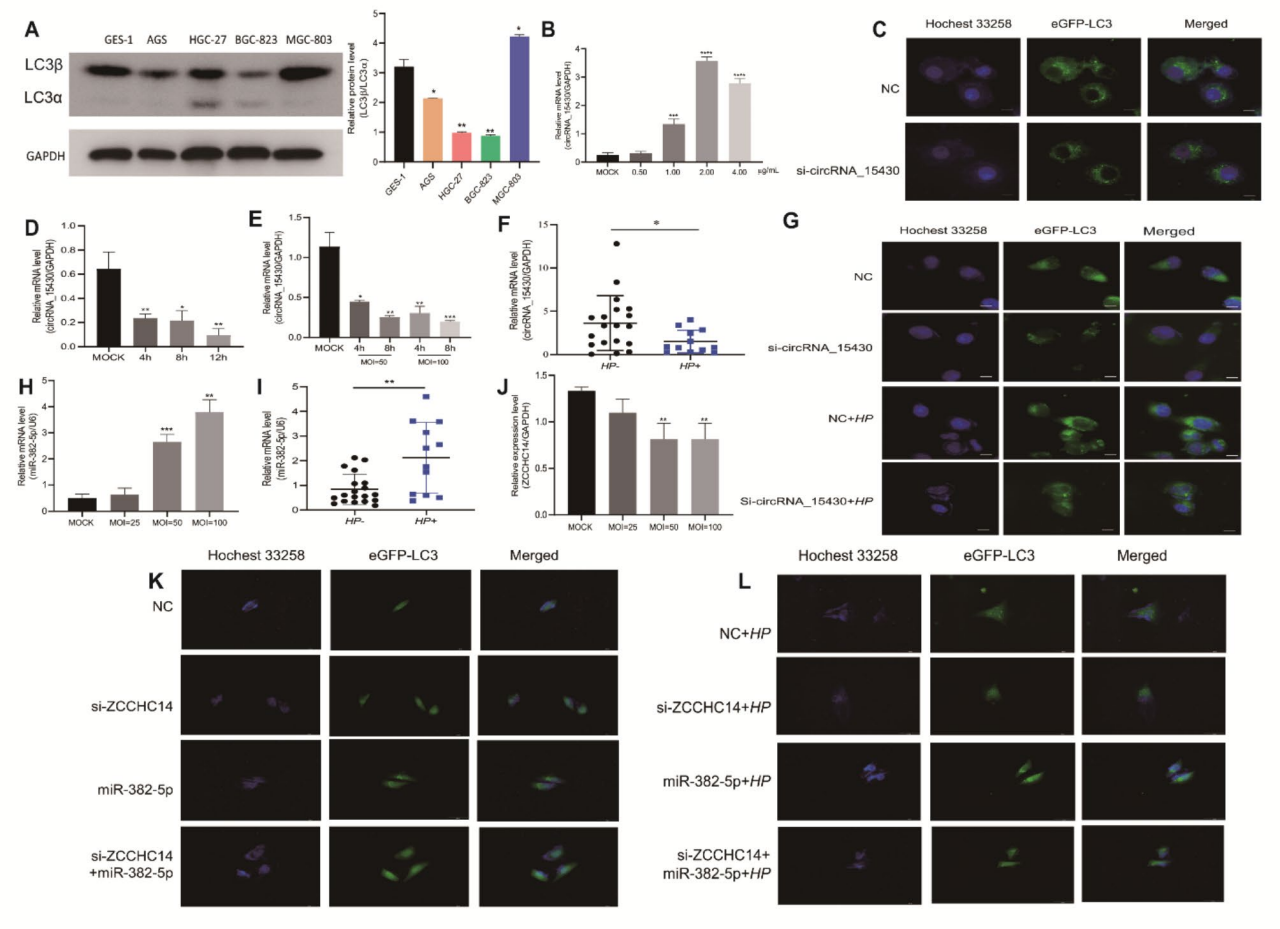
Down-regulation of circRNA_15430 reduced *HP* infection-induced in MGC-803 cells. **A** The expression of LC3 in GES-1 cells and GC cells were detected by Western Blotting **B** The expression of circRNA_15430 was detected by qRT-PCR when MGC-803 cells were treated with different concentrations of Rapamycin. **C** The number of autophagosomes was detected by confocal laser microscopy when circRNA_15430 was knocked down in MGC-803 cells (scale bars = 25 μm). **D** After *HP26695* infected MGC-803 cells at a MOI = 50:1 for different times (0, 4, 8, and 12 h), the relative expression of circRNA_15430 was assessed by qRT-PCR. **E** After *HP26695* infected MGC-803 cells with different MOIs (0, 50 and 100) for 4 and 8 h, the expression of circRNA_15430 was detected by qRT-PCR. **F** qRT-PCR monitored the expression of circRNA_15430 in *HP +* gastritis tissues (n = 12) and *HP-* gastritis tissues (n = 19). **G** confocal laser microscopy analyzed the number of autophagosomes when *HP26695* was infected with the circRNA_15430 knockdown MGC-803 cells (scale bars = 25 μm). **H** The expression level of miR-382-5p in MGC-803 cells infected with *HP* for 12 h with different MOI values (MOI = 25, 50 and 100) by qRT-PCR. **I** The expression level of miR-382-5p in *HP +* gastritis tissues (n = 12) and *HP-* gastritis tissues (n = 19) were detected by qRT-PCR. **J** The expression level of ZCCHC14 in MGC-803 cells infected with *HP* for 12 h with different MOI values (MOI = 25, 50 and 100) by qRT-PCR. **K** The number of autophagosomes was detected by confocal laser microscopy when the expression of ZCCHC14 and miR-382-5p was decreased and **L***HP26695* infected MGC-803 cells for 12 h with MOI = 50 (scale bars = 50 μm). *P < 0.05, **P < 0.01, ***P < 0.001

CircRNA such as circCUL2 and hsa_circ_0009735 may regulate cell autophagy in GC cells [23, 24]. Furthermore, in GES-1 cells and GC cells, the ratio of LC3β/LC3α was highest in MGC-803 cells (Fig. 7A). The expression level of circRNA_15430 increased with increasing rapamycin concentration in MGC-803 cells, which was significant at 1.00 µg/mL (Fig. 7B). Confocal laser microscopy showed that the knockdown of circRNA_15430 in MGC-803 cells could decrease the number of autophagosomes (Fig. 7C). In addition, *HP26695* decreased the expression of circRNA_15430 in MGC-803 cells at a specific time (Fig. 7D) and different MOI values (Fig. 7E), and the expression of circRNA_15430 was much lower in *HP +* chronic gastritis tissues than in *HP-* chronic gastritis tissues (Fig. 7F). Confocal laser microscopy detected and found that *HP* infected MGC-803 cells could increase the number of autophagosomes, and the reduction of circRNA_15430 could reverse the effects of *HP* on autophagy (Fig. 7G). Hence, the knockdown of circRNA_15430 could inhibit *HP* infection-regulated cell autophagy in MGC-803 cells. Furthermore, the expression of miR-382-5p was increased with the increased of MOI value of *HP* infected with MGC-803 cell (Fig. 7H), and it was up-regulated in *HP +* chronic gastritis tissues than in *HP-* chronic gastritis tissues (Fig. 7I). In addition, *HP* infected with MGC-803 cells with different MOI decreased ZCCHC14 expression (Fig. 7J). Furthermore, knockdown of ZCCHC14 in MGC-803 cells reduced the number of autophagosomes and miR-382-5p inhibitor increased the number of autophagosomes (Fig. 7K), then *HP26695* infected MGC-803 cells increased the number of autophagosomes obviously and the number of autophagosomes reduced by ZCCHC14 knockdown can be recovered with miR-382-5p inhibitor (Fig. 7L).

## Discussion

*Helicobacter pylori (HP)* are micro-aerobic colonizing bacteria that can grow and multiply in the stomach. It is the main pathogen of gastric diseases such as chronic gastritis, peptic ulcers, and giant mucosal fold disease, and is closely related to the occurrence of gastric cancer and gastric mucosa-associated lymphoid tissue lymphoma. *HP* was classified as a class I carcinogen by WHO in 1994 [25, 26] and a definite carcinogen by the United States in December 2021. Recently, many studies found that circRNAs play an important role in the development of GC and are expected to become a basic theoretical target for cancer prevention, diagnosis, and treatment [14, 27, 28]. In the present study, circRNA_15430 was reverse spliced in the transcription process of the *abl2* gene. Sequencing of the PCR product showed that it matched with hsa_circ_0015430 and had cyclization sites. The expression of circRNA_15430 was decreased in GC cell lines and tissues and associated with tumor sizes in GC patients while no significant correlated with OS and PFS, the small number of clinical specimens maybe the main reason for this result, and further collection of clinical GC specimens is needed to analyze the relationship between them. Localization analysis showed that circRNA_15430 was present in the nucleus and cytoplasm and mainly in the cytoplasm of GC cells. The knocked down of circRNA_15430 in MGC-803 cells promoted cell proliferation and migration and inhibited cell apoptosis, facilitating the EMT process. The expression of BAX decreased while BCL2 increased. Compared to the Vector group, the HGC-27 cells and BGC-823 cells with the strong expression of circRNA_15430 suppressed cell proliferation and migration and strengthened cell apoptosis; the cellular proteins changed accordingly. Furthermore, knockdown circRNA_15430 could promote MGC-803 cell growth in vivo.

Although studies have found that circRNAs could have miRNA sponging effects, binding with RNA binding proteins, and regulating transcription and translation, the sponge adsorption function of circRNAs is currently the most widely and deeply studied mechanism. CircRNA-ABCB10 could promote GC progression by sponging miR-1915-3p to mediate RaC [29]. Circular RNA circ0007360 attenuates GC progression by altering the miR-762/ IRF7 axis [30]. CircBank, starbase, and circular RNA interactome predicted that miR-382-5p has a binding site with circRNA_15430, and qRT-PCR showed that miR-382-5p expression was opposite that of circRNA_15430. The luciferase reporter assay proved that circRNA_15430 could bind to miR-382-5p and miR-382-5p, acting as cancer-promoting miRNAs in GC cells. MiR-382-5p mimics can reverse the function of OE-circRNA_15430 in the metastasis, proliferation, and apoptosis of GC cells. Subsequently, the public databases and related experiments suggested that ZCCHC14 might be the target gene regulated by miR-382-5p. Also, in GC cells, the expression level of ZCCHC14 was changed with the expression of circRNA15430, knockdown of ZCCHC14 in HGC-27 cells decreases circRNA_15430 expression. Reversed experiments showed that si-RNA of ZCCHC14 could reverse the effects of circRNA_15430 overexpression on metastasis, proliferation and apoptosis of GC cells. Therefore, circRNA_15430 could suppress the progression of GC by regulating the miR-382-5p/ZCCHC14 axis and the mutual regulation between circRNA_15430 and ZCCHC14 affects the progression of GC.

Previous studies explored the role of circRNAs in the autophagy of GC cells [23, 24]. Jiang et al. reported that circ_0032821 was significantly upregulated in human GC tumors and cells, and the knockdown of circ_0032821 decreased cell proliferation, EMT, migration, and invasion but increased the autophagy of AGS and HGC-27 cells in vitro [31]. In addition, circST3GAL6 was downregulated in GC patients, and the overexpression of circST3GAL6 inhibited proliferation and metastasis and induced the apoptosis and autophagy of GC cells [32]. This research showed that the ratio of LC3β/LC3α was highest in MGC-803 cells, in addition, MGC-803 cells treated with rapamycin for 18 h, circRNA_15430 expression increased with increasing concentration, and it was increased significantly at 1.00 µg/mL. After knocking down circRNA_15430 in MGC-803 cells, the number of autophagosomes was significantly lower. The level of circRNA_15430 expression was decreased in *HP +* tissues and *HP-i*nfected MGC-803 cells, and it could reverse the effects of *HP* infection on autophagy. In addition, the expression level of miR-382-5p was increased in *HP +* tissues and *HP-i*nfected MGC-803 cells, while ZCCHC14 was decreases in *HP-i*nfected MGC-803 cells. Also, *HP* infection of MGC-803 cells promoted the generation of autophagosomes, and the decrease in autophagosome number caused by the decrease of ZCCHC14 expression could be restored by the miR-382-5p inhibitor.

The main limitation of the study was the small number of included patients. Although the preferred treatment of GC in the referral tertiary center is surgery, numerous patients were initially treated with radiation therapy or chemotherapy. Larger cohorts of patients involving randomized clinical trials would be a step further in determining the true usefulness of biomarkers in the diagnosis, treatment, and prognosis of GC. Furthermore, maybe we can further analyze the signal pathway role in circRNA_15430 suppressing GC progression.

## Conclusion

In conclusion, this study found that circRNA_15430 was downregulated in GC tissues and *HP +* gastritis tissues, suppressed cell proliferation and migration and promoted cell apoptosis, *HP* infected the associated autophagy by the miR-382-5p/ZCCHC14 axis in GC cells. This provides an experimental basis for exploring the molecular mechanism through which circRNA_15430 suppresses the occurrence and development of GC. It suggested that circRNA_15430 could be an effective therapeutic target for GC and diseases associated with HP infection, and provided a intervention target for GC treatment.

### Electronic supplementary material

Below is the link to the electronic supplementary material.

**Additional file 1: Table S1:** The sequences of the primers

**Additional file 2: Table S2:** Antibodies used in the article

## Data Availability

All data generated or analysed during this study are included in this published article.
